# Visualization and identification of benzylisoquinoline alkaloids in various *nelumbo nucifera* tissues

**DOI:** 10.1016/j.heliyon.2023.e16138

**Published:** 2023-05-19

**Authors:** Chenyang Hao, Wei Yang, Gangqiang Dong, Yuetong Yu, Yan Liu, Jun Zhang, Yongping Zhu, Xiaolu Wei, Sha Chen

**Affiliations:** a Key Laboratory of Beijing for Identification and Safety Evaluation of Chinese Medicine, Institute of Chinese Materia Medica, China Academy of Chinese Medical Sciences, No.16, Nanxiaojie, Dongzhimennei, Beijing 100700, China; b Amway (China) Botanical R&D Center, Wuxi 214115, China

**Keywords:** In situ detection, Metabolomics, Benzylisoquinoline alkaloids, MALDI–MSI, UPLC-QTOF-HRMS

## Abstract

Benzylisoquinoline alkaloids in lotus (*Nelumbo nucifera*) seed plumules and leaves exhibit significant tissue specificity for their pharmacological effects and potential nutritional properties. Herein, 46 benzylisoquinoline alkaloids were identified via UPLC-QTOF-HRMS, of which 9 were annotated as glycosylated monobenzylisoquinoline alkaloids concentrated in the seed plumules. The spatial distribution of targeted benzylisoquinoline alkaloids in leaves, seed plumules, and milky sap was determined via MALDI–MSI. Furthermore, 37 *Nelumbo* cultivars were investigated using targeted metabolomics to provide insights into functional tea development. While aporphine alkaloids comprised the main compounds present in lotus leaves, bisbenzylisoquinoline alkaloids were the main compounds in lotus plumules, where glycosylation primarily occurs. These findings can help understand the distribution of benzylisoquinoline alkaloids in lotus tissue and the directional breeding of varieties enriched with specific chemical functional groups for nutritional and pharmacological applications.

## Highlights

• Using UPLC-QTOF-HRMS^n^, 46 alkaloids were identified in lotus.
• Among the 46 alkaloids, 9 were glycosylated monobenzylisoquinolines.
• Their tissue distribution was investigated using MALDI-MSI.
• Natural monobenzylisoquinoline alkaloid glycosylation enriched the glycosylated alkaloids in a tissue-specific manner.
• Our findings provide insights into the biosynthesis of benzylisoquinoline alkaloids.

## 1 Introduction

Sacred lotus (*Nelumbo nucifera* Gaertn.) is a basal, economic crop that is used for both medicinal and food application. Lotus seeds and underground stems are frequently consumed in East and Southeast Asian countries, owing to their high nutritional properties. Lotus leaf extracts possess antiviral, diuretic, and astringent activities and are also used as an antipyretic [1]. Lotus seeds reportedly have anticancer, antidepressant, antioxidant, and anti-inflammatory properties [2,3]. Furthermore, various secondary metabolites in lotus leaves and seeds exhibit tissue specificity and are used in different functional and nutritional [4]. Therefore, understanding the biosynthesis, transportation, and distribution of these metabolites can facilitate to their development and utilization as functional foods [5].

Reportedly, benzylisoquinoline alkaloids (BIAs) constitute the chief components in sacred lotus and possess specialized nutritional value; however, they probably play a key role in defense against herbivores and pathogens in the plant [6]. BIAs constitute a large class of plant specialized metabolites derived from tyrosine [7]. Studying the synthesis, transportation, and distribution of such metabolites can greatly facilitate the directed breeding of varieties enriched with specific functional groups. The genome of the China Antique variety of the sacred lotus has been sequenced and assembled [8]. Over the previous decades, there have been increasing efforts and research interest in studying the biosynthesis and bioavailability of these specialized alkaloids, such as nuciferine, *N*-nornuciferine, armepavine, and neferine [9]. BIA metabolism in lotus has been presumed based on the biosynthetic genes and enzymes involved in the generation of benzylisoquinoline in the plant [10]. Further BIA studies can provide new findings and contribute to the development and application of nutritional food resources.

Mass spectrometry imaging (MSI) has emerged as a powerful analytical technique that can assess the spatial distributions of numerous compounds in biological tissues [11,12]. This technique has been extensively used to determine the spatial distribution of metabolites involved in plant biosynthesis [13,14]. Ultrahigh-performance liquid chromatography–ion-trap high-resolution time-of-flight mass spectrometry (UPLC-QTOF-HRMS) is suitable for analyzing structurally complex natural products. Furthermore, the combined application of MSI and UPLC-QTOF-HRMS has been employed to analyze the synthesis, biosynthesis, and distribution of specialized metabolites in licorice and hypericum [15,16].

This study aimed to investigate the biosynthesis and distribution of different BIAs in various lotus tissues. This analysis comprised four steps: 1) a UPLC-QTOF-HRMS analysis of the chemical profiles of BIAs in the leaves, seed plumules, and milky sap of *Nelumbo*; 2) matrix-assisted laser desorption and ionization MSI (MALDI–MSI) was used to characterize the spatial distribution of BIAs in these three tissues; 3) quantitative UPLC-QTOF-MS analysis of BIAs in 37 *Nelumbo* cultivars; and 4) elucidation of the putative biosynthetic pathways of BIAs. In addition, this study aimed to optimize the application of medicinal plants and promote the development of food resources based on tissue-specific germplasm resources.

## 2 Materials and methods

### 2.1 Reagents and chemicals

Coclaurine (wkq20071007) was purchased from Sichuan Victory Biological Technology Co., Ltd. (Sichuan, China). *N*-nornuciferine (B21944) was obtained from Shanghai Yuanye Bio-technology Co., Ltd. (Shanghai, China). Neferine (BCY-000435), liensinine (BCY-000438), armepavine (BCY-001299), and isoliensinine (250117-201812) were procured from Jiangxi Baicaoyuan Bio-technology Co., Ltd. (Jiangxi, China). Nuciferine (CFN99733) was purchased from Chemfaces. Acetonitrile, methanol, and formic acid (high-performance liquid chromatography grade) were obtained from Sigma-Aldrich Corporation (St. Louis, MO, USA). Ultrapure water was prepared using a Mill-Q SP system (Millipore Co., Bedford, MA, USA).

### 2.2 Plant materials and sample preparation

The seeds of 37 *N. nucifera* (sacred lotus) cultivars were collected from China (Yunnan, Jiangsu, and HuBei Provinces), Japan, and Thailand and identified using previously described morphological and histological methods [17]. Subsequently, these cultivars were grown in the United Lotus Germplasm Resource of the Amway Botanical Research Center (Wuxi, China) and collected in August (Supplementary Table 1). For each germplasm, three replicates of three different lotus seeds were used.

Subsequently, 0.5 mL of fresh milky sap (obtained from *Nelumbo* stem) was extracted using 3 mL of extract solution (0.3 M hydrochloric acid:methanol, 1:1, v/v) via sonication for 20 min followed by centrifugation at 12,000 rpm for 3 min. Each sample extraction was repeated twice, and the supernatant was collected and filtered using a 0.22-μm Millipore filter (Alltech Scientific Corporation, Beijing, China) before UPLC-mass spectrometry (UPLC-MS/MS). Next, 0.5 g of fresh seed plumules and leaves was accurately weighed and then powdered in liquid nitrogen using an analytical mill (IKA A11 basic machine, Germany). The samples were extracted in a 6-mL mixed solution (0.3 M HCl: methanol, 1:1; v/v) for 20 min via ultrasonication followed by centrifugation at 5000×*g* for 10 min, and the supernatant was filtered using a 0.22-μm filter (Alltech Scientific Corporation, Beijing, China) before UPLC-MS/MS analysis [18,19]. Then, 60 μL of a methanol:water, (1:1, v/v) solution was added to the milky sap and centrifuged at 12,000×*g* for 5 min, followed by the removal of 0.5 μL of supernatant. An equal volume of 2,5-dihydroxybenzoic acid solution was added to the mixed well, smeared onto a slide, dried, and analyzed using MSI.

The fresh leaf and seed plumules were mixed with 5% carboxymethyl cellulose solution and stored in Tissue-Tek® molds. Cryosections of 16-μm thickness were obtained at −20 °C and mounted on glass slides for MSI measurements.

### 2.3 MALDI-MSI

All measurements were performed using AP-SMALDI 10 high-resolution MALDI imaging ion source (TransMIT GmbH, Giessen, Germany), which was operated at atmospheric pressure and coupled to a Q-Exactive Orbitrap mass spectrometer (Thermo Fisher Scientific, Bremen, Germany). The ion source is a solid laser (λ = 343 nm) operating at a repetition rate of 2000 Hz. For each mass spectrum, ions from 50 laser pulses were accumulated in the C-trap before being sent to the Orbitrap mass analyzer. All the experiments were performed in the positive-ion mode with the target voltage at +4.3 kV.

MSI of the lotus leaf, seed plumule and milky sap sections was performed at 15, 30, and 30 μm spatial resolutions in areas of 4290 × 795 μm^2^ (286 × 53 pixels), 3210 × 4110 μm^2^ (107 × 137 pixels) and 1380 × 1590 μm^2^ (46 × 53 pixels), respectively. The measurement speed in the full scan mode (scan range *m*/*z* 100–700) was ∼1.5 s/pixel at a mass resolution of 70,000 @ *m*/*z* 200. The step size of the sample stage was the desired pixel size.

### 2.4 Liquid chromatography–mass spectrometry analysis

Agilent UPLC 1290II system combined with a 6540 QTOF (Agilent Technologies, Santa Clara, CA, USA) was used to determine the accurate mass of the metabolites. UPLC equipped with a binary solvent delivery system, autosampler, and column compartment was used in this study. Chromatographic separation was performed on a Waters BEH C_18_ column (2.1 × 100 mm, 1.7 μm), and the elution conditions were as follows: 0–15 min, 5%–95% B. A and B indicate 0.1% formic acid water (formic acid:water, 0.1:100, v/v) and acetonitrile, respectively.

Sample ionization was acquired in both positive and negative modes within the mass/charge (*m*/*z*) range of 50–1000. The electrospray ionization (ESI) source operating parameters in both the positive and negative modes and the ESI–MS conditions were as follows: gas temperature, 325 °C; gas flow, 5 L/min; nebulizer, 35 psig; and sheath gas temperature, 350 °C. Internal references (purine and HP-0921) were adopted to modify the measured masses in real time, and the reference masses were *m/z* 121.0509 and 922.0098 in the positive-ion mode and 119.0363 and 1033.9881 in the negative-ion mode. The accurate mass of each metabolite was used for quantification.

### 2.5 Statistical analysis

Origin 2021b for Windows® (Origin Lab Corp., USA) was used to calculate the correlation coefficients of the alkaloids in the various tissues. Principal component analysis (PCA) was performed using SIMCA software version 14.1 (Umetrics, Sweden) to elucidate the differences between the four types of alkaloids detected in the three different tissue types.

## 3 Results and discussion

### 3.1 Metabolic profiling of BIAs in lotus

Overall, 46 alkaloids were identified in the leaves, seed plumules, and milky sap of lotus. The accurate masses, fragmentation ions of MS/MS, and retention times of 19, 25, and 30 alkaloids were identified in lotus leaves, lotus plumules, and milky sap, respectively, were determined. (Table 1).

**Table 1:** Identification of alkaloids in lotus leaf (LL), lotus milky sap (LMS), and lotus plumule (LP).

NO.	Rt (min)	[M+H]^+^	Molecular formula	Error (ppm)	Fragmentation profile (*m*/*z*) (Only high relative abundance %)	Compound identification	Tissue
Nineteen monobenzylisoquinoline alkaloids (LL 10, LP 15, LMS 9)							
1	2.76	272.1289	C_16_H_17_NO_3_	2.94	107.0496 (100), 161.0606 (44.06), 255.1023 (34.55)	Norcoclaurine	LL, LMS
2	2.81	286.1424	C_17_H_19_NO_3_	−4.89	107.0498 (100), 286.1451 (92.88), 255.1027 (51)	*N*-methylhigenamine	LL, LP, LMS
3	3.48	286.1446	C_17_H_19_NO_3_	2.80	107.0497 (100), 269.1171 (63.26), 237.092 (54.51),	Isococlaurine	LL, LMS
4^b^	4.38	286.1437	C_17_H_19_NO_3_	−0.35	269.1194 (100), 107.0491 (96.39)	Coclaurine	LL, LP, LMS
5	3.57	300.1605	C_18_H_21_NO_3_	3.66	107.0497 (100), 269.1176 (72.02), 175.076 (28.12)	*N*-methylisococlaurine	LL, LP, LMS
6	4.19	300.1604	C_18_H_21_NO_3_	3.33	107.0495 (100), 283.1336 (96.75), 189.0912 (33.12)	Norarmepavine	LL
7	4.38	300.1598	C_18_H_21_NO_3_	1.33	300.1576 (100), 107.0487 (49.83), 269.1147 (38.84), 175.0732 (18.73)	*N*-methylcoclaurine	LP
8^b^	4.16	314.1741	C_19_H_23_NO_3_	−3.18	283.1326 (100), 107.049 (78.75), 189.0908 (65.2)	Armepavine	LL, LP, LMS
9	3.02	314.1762	C_19_H_24_NO_3_	3.50	58.0658 (100), 269.1178 (18.22), 107.0497 (15.01)	Lotusine	LL, LP, LMS
10^a^	3.74	328.1915	C_20_H_25_NO_3_	2.44	328.1909 (100), 58.0658 (74), 283.1331 (38.14), 107.0493 (24.67)	4′-*O*-Methylarmepavine	LP
11^a^	1.14	448.1903	C_23_H_29_NO_8_	−4.6	286.142 (100), 448.1907 (79.8), 107.0692 (17.92), 255.0908 (17)	*N*-methylhigenamine-7-*O*-Glc	LP
12^a^	2.11	448.1917	C_23_H_29_NO_8_	−4.9	448.1912 (100), 286.1377 (44.53), 255.0992 (12.41), 107.0487 (6.8)	*N*-methylhigenamine-4′-*O*-Glc	LP
13^a^	2.38	448.1976	C_23_H_29_NO_8_	2.23	286.1398 (100), 448.1916 (76.68), 255.0967 (22.53), 107.0483 (14.2)	*N*-methylhigenamine-6-*O*-Glc	LP, LMS
14^a^	2.69	448.1975	C_23_H_29_NO_8_	2.01	448.1933 (100), 286.1383 (98.25), 269.1144 (52.93), 107.0494 (27.79)	Isococlaurine-Glc	LP
15^a^	3.55	448.1989	C_23_H_29_NO_8_	3.13	448.1893 (100), 286.1407 (49.99), 269.1113 (34.34), 107.0492 (9.56)	Coclaurine-Glc	LL, LP
16^a^	2.48	462.2124	C_24_H_31_NO_8_	0.43	462.212 (100), 300.1584 (38.1), 269.1164 (11.8)	*N*-methylisococlaurine-4′-*O*-Glc	LP
17^a^	2.72	462.2136	C_24_H_31_NO_8_	3.03	462.206 (100), 300.1555 (28.77), 269.1132 (12.24), 107.0475 (7.17)	*N*-methylisococlaurine-6-*O*-Glc	LP, LMS
18^a^	3.35	462.2113	C_24_H_31_NO_8_	−1.95	236.011 (100), 300.1495 (48.37), 283.1263 (12.64)	Norarmepavine-Glc	LL
19^a^	3.55	462.2144	C_24_H_31_NO_8_	4.76	462.2055 (100), 300.1544 (33.33), 269.105 (5.61)	*N*-methylcoclaurine-4′-*O*-Glc	LP
Ten apophine alkaloids (LL 9, LP 7, LMS 8)							
20	2.51	264.1062	C_17_H_13_NO_2_	4.28	101.006 (100), 131.9873 (57.1), 152.0242 (24.18)	Dehydroanonaine	LL, LMS
21	5.59	266.1186	C_17_H_15_NO_2_	3.76	249.0916 (100), 219.0807 (27.79), 191.0858 (17.18)	Anonaine	LL, LP, LMS
22	4.60	268.1337	C_17_H_17_NO_2_	1.86	219.0805 (100), 191.0864 (62.39), 251.1077 (37.73)	Caaverine	LL, LMS
23	5.81	280.1342	C_18_H_17_NO_2_	3.57	249.0913 (100), 219.08 (17.91)	Roemerine	LL, LP, LMS
24	4.75	282.1487	C_18_H_19_NO_2_	−0.71	251.1071 (100), 219.0819 (55.98), 191.0869 (32.01)	*O*-nornuciferine	LL, LP, LMS
25	5.07	282.1489	C_18_H_19_NO_2_	0.00	251.1044 (100), 219.0781 (32.26), 191.0128 (7.58)	Lirinidine	LP
26^b^	5.75	282.15	C_18_H_19_NO_2_	3.90	265.1227 (100), 250.0992 (64.23), 234.1045 (32.83)	*N*-nornuciferine	LL, LP, LMS
27^b^	5.86	296.1655	C_19_H_21_NO_2_	3.38	265.1234 (100), 250.0995 (43.81), 234.1048 (22.68)	Nuciferine	LL, LP, LMS
28	2.67	298.1446	C_18_H_19_NO_3_	2.68	255.1011 (100), 223.077 (57.7)	Glaziovine	LL
29	3.69	312.16	C_19_H_21_NO_3_	1.92	269.1181 (100), 254.0939 (36.95), 283.1335 (35.49), 206.1182 (35.41)	Pronuciferine	LL, LP, LMS
Four bisbenzylisoquinoline alkaloids (LL 0, LP 3, LMS 4)							
30	3.45	597.2982	C_36_H_40_N_2_O_6_	3.85	597.2967 (100), 192.1044 (86.83), 475.2278 (26.66)	Nelumboferine	LMS
31^b^	3.83	611.3122	C_37_H_42_N_2_O_6_	0.98	611.3119 (100), 206.1176 (71.96), 489.2418 (30.64), 568.2706 (19.38), 580.2682 (12.78)	Liensinine	LP, LMS
32^b^	4.07	611.3118	C_37_H_42_N_2_O_6_	0.33	611.3083 (100), 192.1006 (80.07), 475.2186 (22.78)	Isoliensinine	LP, LMS
33^b^	4.52	625.3288	C_38_H_44_N_2_O_6_	2.56	625.331 (100), 206.1179 (94.49), 489.2244 (83.14), 594.2837 (49.7), 582.2859 (30.99)	Neferine	LP, LMS
Thirteen other alkaloids (LL 9, LP 7, LMS 10)							
34^a^	1.31	118.0870	C_5_H_11_NO_2_	4.93	59.0497 (100)	Betaine	LL, LMS
35^a^	2.12	120.0806	C_8_H_9_N	−1.67	77.0393 (100), 103.0547 (61.99), 91.0546 (41.85)	*N*-Benzylidenemethylamine	LL, LP, LMS
36^a^	14.52	326.3063	C_20_H_39_NO_2_	2.76	62.0598 (100)	*N*-Oleoylethanolamine	LL, LP, LMS
37^a^	1.48	138.0915	C_8_H_11_NO	1.45	77.0378 (100), 103.0528 (57.95), 121.0266 (43.91)	Tyramine	LP
38^a^	1.71	150.0559	C_8_H_7_NO_2_	6.00	61.0294 (100), 98.9764 (24.69)	4-Hydroxymandelonitrile	LL, LP, LMS
39^a^	1.39	182.0816	C_9_H_11_NO_3_	2.20	91.0544 (100), 123.044 (57.76), 136.0768 (57.49), 77.0388 (29.05)	l-Tyrosine	LL, LP
40^a^	1.05	138.0553	C_7_H_7_NO_2_	2.17	138.055 (100), 94.0654 (92.94), 92.0506 (87.36)	Trigonelline	LL, LP, LMS
41	1.47	144.1015	C_7_H_13_NO_2_	−2.78	55.0547 (100), 84.0812 (70.32)	Stachydrine/N,N-Dimethyl-l-proline	LL
42^a^	5.80	267.1244	C_17_H_16_NO_2_	−3.74	249.0907 (100), 251.103 (40.53), 266.127 (39.94)	Vasconine	LL, LMS
43	1.77	118.0638	C_8_H_7_N	−4.9	77.0396 (100), 59.0498 (88.6), 91.0505 (52.34)	Indole	LMS
44^a^	1.31	154.0869	C_8_H_11_NO_2_	3.89	91.0546 (100), 137.0602 (50.12), 119.0496 (30.76)	Dopamine	LMS
45^a^	1.71	177.1032	C_10_H_12_N_2_O	5.65	160.0765 (100), 132.0813 (14.89), 115.0564 (11.76)	Serotonin	LP, LMS
46^a^	2.84	205.0969	C_11_H_12_N_2_O_2_	−1.46	146.06 (100), 118.0653 (55.08)	Tryptophan	LL, LMS

LL: lotus leaf; LP: lotus plumule; LMS: lotus milky sap.

a Alkaloids reported in lotus for the first time.

b Compounds identified using standards.

The accurate masses of the metabolites were determined by performing a full scan using QTOF-HRMS. The available fragmentation profiles of the compounds were obtained via product ion or a neutral loss scan. Compounds coclaurine **4**, armepavine **8**, *N*-nornuciferine **26**, nuciferine **27**, liensinine **31**, isoliensinine **32**, and neferine **33** were identified by matching the MS and MS/MS spectra to the authentic reference standards.

#### 3.1.1 Monobenzylisoquinoline alkaloids

In total, 19 monobenzylisoquinoline alkaloids were identified; among them, 10, 15, and 9 were quantified in lotus leaves, seed plumules, and lotus milky sap, respectively.

Compound **1** was identified as norcoclaurine, with a molecular formula of C_16_H_17_NO_3_ and characteristic loss ions of C_7_H_7_O *m/z* 107.0496 and [M + H–C_7_H_11_O]^+^ *m/z* 161.0606 produced by benzyl-group breaks and a neutral loss ion of [M + H–NH_3_]^+^ *m/z* 255.1023. Based on the molecular formula C_20_H_25_NO_3_ and a characteristic loss ion of [M + H–C_7_H_11_O]^+^ *m/z* 107.0493, **10** was tentatively identified as 4′-*O*-methylarmepavine.

**Fig. 1:**
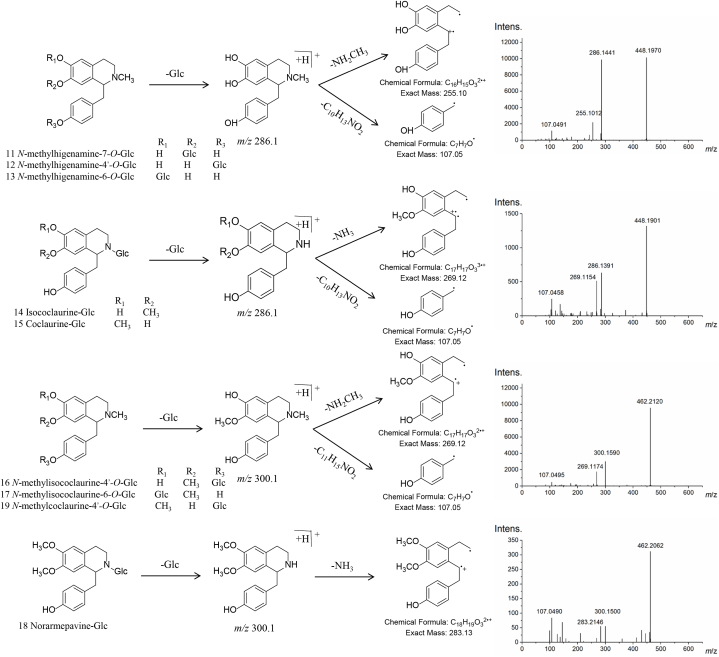
The mass spectrometry fragmentation and the identification of nine alkaloid glycosides in lotus using UPLC-QTOF-HRMS.

The most common modification reaction of the metabolites was glycosylation, which increases the solubility and stability of compounds [20]. Glucoside (Glc)- substituted compounds were common in lotus. Compounds **11**–**19** were deduced to have undergone glycosylation with monobenzylisoquinoline alkaloids. Five alkaloid compounds (**11**–**15**) exhibited the same molecular formula of C_23_H_29_NO_8_ and [M + H-162Da]^+^ characteristic ion, indicating loss of glucoside and fragment ions [M + H-162Da-NH_3_]^+^ *m/z* 255.0992 and [M + H–C_7_H_11_O]^+^ *m/z* 107.0692. Compounds **11**–**13** were predicted as *N*-methylhigenamine-7-*O*-Glc, *N*-methylhigenamine-4′-*O*-Glc, *N*-methylhigenamine-6-*O*-Glc, respectively (Fig. 1). The MS/MS fragmentation of compounds **14** and **15** afforded significant characteristic ions [M + H-162Da-NH_3_]^+^ *m/z* 269.1114 and [M + H–C_7_H_7_O]^+^ *m/z* 107.0492, and consequently, the compounds were annotated as isococlaurine-Glc and coclaurine-Glc, respectively, based on the compound identification database (MassBank, http://www.massbank.jp/Search) (Fig. 1). Four monobenzylisoquinoline alkaloids (**16**–**19**) were deduced to have the same molecular formula C_24_H_31_NO_8_ and fragment ion [M + H-162Da]^+^ *m/z* 300.1584. Owing to the characteristic ion [M + H-162Da-NH_3_]^+^ *m/z* 283.1263, **18** was tentatively identified as norarmepavine-Glc. Because of the fragment ions [M + H-162Da-CH_3_NH_2_]^+^ *m/z* 269.1132 and [M + H–C_7_H_7_O]^+^ *m/z* 107.0475, **17** was speculated as *N*-methylisococlaurine-6-*O*-Glc. Compound **16** was annotated as *N*-methylisococlaurine-4′-*O*-Glc with the same MS/MS spectral behavior of **17**. Compound **19** was predicted as *N*-methylcoclaurine-4′-*O*-Glc (Fig. 1).

Three monobenzylisoquinoline alkaloids (compounds **2**–**4**) exhibited the protonated ion [M+H]^+^ at *m/z* 286.1446. In the MS/MS spectra of **3** and **4**, the fragment ions [M + H–C_7_H_11_O]^+^ *m/z* 107.0497, [M + H–NH_3_]^+^ *m/z* 269.1171, [M + H–NH_3_–CH_3_OH]^+^ *m/z* 237.092, and [M + H–C_7_H_11_O]^+^ *m/z* 175.0497 were observed; therefore, these compounds were identified as isococlaurine and coclaurine, respectively, and this has been confirmed using the authentic standards. Furthermore, the elution times of the substituent C-7 alkaloids were longer than those of C-6 alkaloids on C_18_ columns; thus confirming the identities of **3** and **4**, which has been previously reported in lotus plumules [21]. In the MS/MS spectra of **2**, the ion [M + H–CH_5_N]^+^ *m/z* 255.1027 was detected, hence, **2** was determined to be *N*-methylhigenamine.

Three monobenzylisoquinoline alkaloids (**5**–**7**) exhibited the same molecular formula C_18_H_19_NO. Compound **6** was identified as norarmepavine, with fragment ions [M + H–NH_3_]^+^ *m/z* 283.1326 and [M + H–C_7_H_11_O]^+^ *m/z* 189.0912 in the MS/MS spectra. The same characteristic fragments [M + H–CH_3_NH_2_]^+^ *m/z* 269.1176 and [M + H–C_8_H_13_O]^+^ *m/z* 175.0760 were observed in the MS/MS spectra of **5** and **7**, and considering the elution order on C_18_ columns, the two compounds were identified as *N*-methylisococlaurine and *N*-methylcoclaurine, respectively [21]. In the MS/MS spectra of **8** and **6**, the characteristic ions [M + H–CH_3_NH_2_]^+^ *m/z* 283.1326, [M + H–C_7_H_7_O]^+^ *m/z* 107.0495 were observed, and according to authentic standards, **8** was identified as armepavine. With the characteristic fragments *m/z* 58.0658, [M + H–NH_3_]^+^ *m/z* 269.1178, and [M + H–C_7_H_7_O]^+^ *m/z* 107.0497, **9** was identified as lotusine.

Compound **10** exhibited the protonated ion [M+H]^+^ *m/z* 328.1915 and was deduced to have the molecular formula C_20_H_25_NO_3_. According to the database (MassBank, http://www.massbank.jp/Search), **10** was tentatively identified as 4′-*O*-methylarmepavine via comparison with the ion peak intensity of [M + H–C_3_H_8_N]^+^ *m*/*z* 58.0658 and [M + H–C_7_H_7_O]^+^ *m*/*z* 107.0493.

#### 3.1.2 Aporphine alkaloids

In total, 10 aporphine alkaloids were identified in *N. nucifera*; among them, **21**, **23**, **24**, **26**, **27**, and **29** were identified and quantified in lotus leaves, lotus milky sap, and seed plumules. For typical aporphines, the substitution of methoxy (OCH_3_) or hydroxyl occurs mainly in the A ring, and major methyl substitution occurs in the ***N* linkage** [22]. If it is an adjacent methoxy and hydroxyl substitution, there are abundant fragment ions due to the loss of CH_3_OH and CO. Because of α cracking that usually occurs at the *N* groups, the loss of NH_3_ and CH_3_NH_2_ fragments could be present in the MS of aporphine alkaloids.

For typical aporphines, the Retro Diels–Alder reaction usually occurs in the B ring via the loss of a CH_2_=N-R group. Compound **29** was deduced to have a molecular formula of C_19_H_21_NO_3_, and the fragment ions [M + H-(CH_2_=NCH_3_)]^+^ *m/z* 269.1181, [M + H-(CH_2_=NCH_3_–CH_3_)]^+^ *m/z* 254.0939, and [M − CO]^+^ *m/z* 283.1335; hence, it was identified as pronuciferine according to a previous report [23]. Compound **28** exhibited the protonated ion [M + H-(CH_2_=NCH_3_)]^+^ *m/z* 255.1011 and [M + H-(CH_2_=NCH_3_)–OCH_3_]^+^ *m/z* 223.077, and hence, it was tentatively identified as glaziovine, which has been previously reported in lotus leaf stalks [24].

#### 3.1.3 Bisbenzylisoquinoline alkaloids

Two bisbenzylisoquinoline alkaloid compounds, **31** and **32**, had the same molecular formula, C_37_H_42_N_2_O_6_. The MS/MS spectrum of the precursor ion *m/z* 611.3116 exhibited intensive characteristic fragments. Compound **31** was tentatively identified as liensinine with fragments ions [M + H–C_25_H_26_NO_4_]^+^ *m/z* 206.1176 and [M + H–CH_3_–CH_2_–C_6_H_5_O]^+^ *m*/z 489.2148, which was confirmed with the authentic standards. Because of the fragment ions [M + H–C_26_H_28_NO_4_]^+^ *m/z* 192.1006 and [M + H–CH_3_–CH_2_–CH_2_–C_6_H_5_O]^+^ *m/z* 475.2186, **32** was unequivocally identified as isoliensinine, which was confirmed according to the authentic standards. In the MS/MS spectra of **33** and **31**, the ions [M + H–C_26_H_28_NO_4_]^+^ *m/z* 206.1179 and [M + H–CH_3_–CH_2_–CH_2_–C_6_H_5_O]^+^ *m/z* 489.2244 were observed; therefore, **33** was unequivocally identified as neferine based on a previous report [25] and the authentic standards. Compound **30** was identified as nelumboferine based on the molecular formula C_36_H_40_N_2_O_6_ and fragment ions [M + H–C_26_H_28_NO_4_]^+^ *m/z* 192.1044 and [M + H–CH_3_–CH_2_–C_6_H_5_O]^+^ *m/z* 475.2186.

#### 3.1.4 Other alkaloids

Thirteen other alkaloids, including amine, pyridine, isoquinoline, and indole alkaloids, were identified in lotus leaves, milky sap, and seed plumules. Overall, 21 alkaloids were identified in the sacred lotus, including 9 glycosylated monobenzylisoquinoline alkaloids. Seven glycosylated monobenzylisoquinoline alkaloids were detected in lotus seed plumules, indicating it as the primary site of glycosylation.

### 3.2 In situ detection of BIAs in lotus

**Fig. 2:**
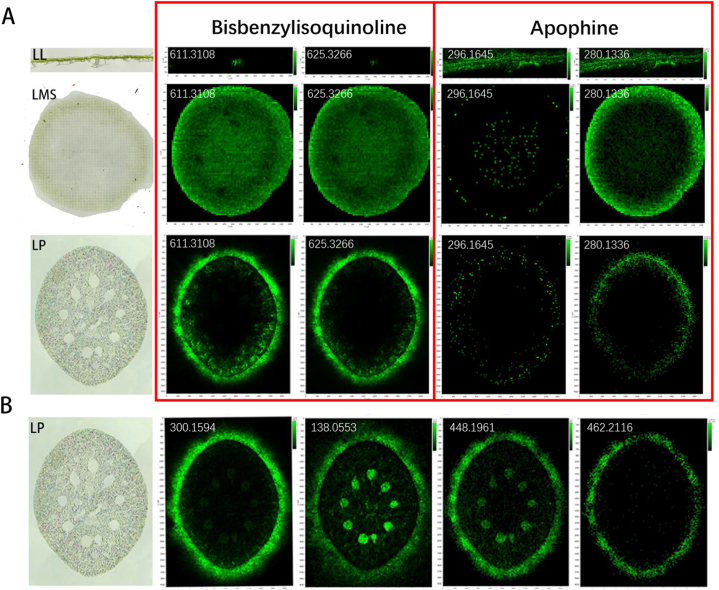
Detection of benzylisoquinoline alkaloids in lotus leaves, milky sap, and seed plumules using MALDI–MSI. (**A)** Distribution and normalized relative abundance of liensinine/isoliensinine ([M+H]^+^, *m/z* 611.3108), neferine ([M+H]^+^, *m/z* 625.3266), and nuciferine ([M+H]^+^, *m/z* 296.1645), roemerine ([M+H]^+^, *m/z* 280.1336) in three tissues (LL, lotus leaf; LMS, lotus milky sap; LP, lotus seed plumules). (**B)** Tissue-specific distribution of methylation and glycosylation in Lps. 4′-*O*-methylarmepavine ([M+H]^+^, *m/z* 328.1915), trigonelline ([M+H]^+^, *m/z* 138.0553), *N*-methylhigenamine-7-*O*-Glc/N-methylhigenamine-4′-*O*-Glc/N-methylhigenamine-6-*O*-Glc/Isococlaurine-Glc/Coclaurine-Glc ([M+H]^+^, *m/z* 448.1961), and *N*-methylisococlaurine-4′-*O*-Glc/*N*-methylisococlaurine-6-*O*-Glc/*N*-methylcoclaurine-4′-*O*-Glc/Norarmepavine-Glc ([M+H]^+^, *m/z* 462.2116).

The distribution of alkaloids in lotus leaf, lotus milky sap, and lotus plumules was studied using MALDI–MSI. The nutritional applications of lotus were associated with the visualization results of the BIAs. The distribution of specific BIAs in different tissues as shown via MSI can benefit the commercial production alongside clinical applications of lotus. BIAs exhibited obvious specific tissue distributions, which supported the speculation that different BIAs were synthesized in the different tissues of lotus (Fig. 2). Combining these findings with those of fluorescence intensity in QTOF MS, we concluded that the typical bisbenzylisoquinoline compounds, such as liensinine/isoliensinine (**31/32**; [M+H]^+^ *m/z* 611.3108) and neferine (**33**; [M+H]^+^ *m/z* 625.3266) were concentrated and distributed in lotus seed plumules and milky sap (Fig. 2A). Seven aporphine alkaloids were detected in the three tissues; however, their contents were the lowest in the seed plumules (Fig. 2A). The results also suggested that *O*-nornuciferine/*N*-nornuciferine/lirinidine (**24/26/25**; [M+H]^+^ *m/z* 282.1489) and dehydroanonaine (**20**; [M+H]^+^ *m/z* 264.1062) could be generated in leaves, which has been previously reported in lotus [26]. Moreover, pronuciferine (**29**; [M+H]^+^ *m/z* 312.1599) may be produced by laticifers and transferred to other tissues. It was apparent that very small amounts of bisbenzylisoquinoline were distributed in the center of lotus leaves and widely distributed in the milky sap, primarily around the seed plumules, suggesting that latex affected its transportation, similar to that in opium poppy. Nuciferine (**27**; [M+H]^+^ *m/z* 296.1645) has been previously reported to be the most important alkaloid in all the growth and development stages of lotus leaves [27], and to be only widely distributed in lotus leaves. Similarly, high levels of roemerine (**23**) were detected in lotus leaves and milky sap. Hence, it can be concluded that the metabolism of aporphine and bisbenzylisoquinoline alkaloids are different in various lotus tissues, which has been previously reported [28]. However, some of the compounds were produced during the growth and development of the tissues. Laticifers and milky sap have an indispensable effect on the generation and transportation of these compounds for stress resistance [5].

The specificity distribution of methylation and glycosylation in the seed plumule is shown in Fig. 2B. Notably, glycosylated monobenzylisoquinoline alkaloids were detected not only around the seed plumules but also in the vascular bundle. The distribution of glycosylated monobenzylisoquinoline alkaloids, including *N*-methylisococlaurine-4′-*O*-Glc, *N*-methylisococlaurine-6-*O*-Glc, *N*-methylcoclaurine-4′-*O*-Glc, and norarmepavine-Glc (**16/17/19/18**), has the same enrichment rules as nonglycosylated monobenzylisoquinoline alkaloids. Five compounds, *N*-methylhigenamine-7-*O*-Glc, *N*-methylhigenamine-4′-*O*-Glc, *N*-methylhigenamine-6-*O*-Glc, and isococlaurine-Glc/Coclaurine-Glc (**11/12/13/14/15**), were detected in the same position, suggesting that these four compounds might be involved in monobenzylisoquinoline glycosylation. The distribution of bisbenzylisoquinoline and aporphine alkaloids differed between leaves and plumules. The areas of glycosylation with benzylisoquinolines were determined using MSI. Additionally, the discovery of trigonelline as a hormone associated with seeding supports the glycosylation process of alkaloids [29,30].

### 3.3 UPLC-QTOF-MS–based metabolomics analysis of BIAs in lotus

**Fig. 3:**
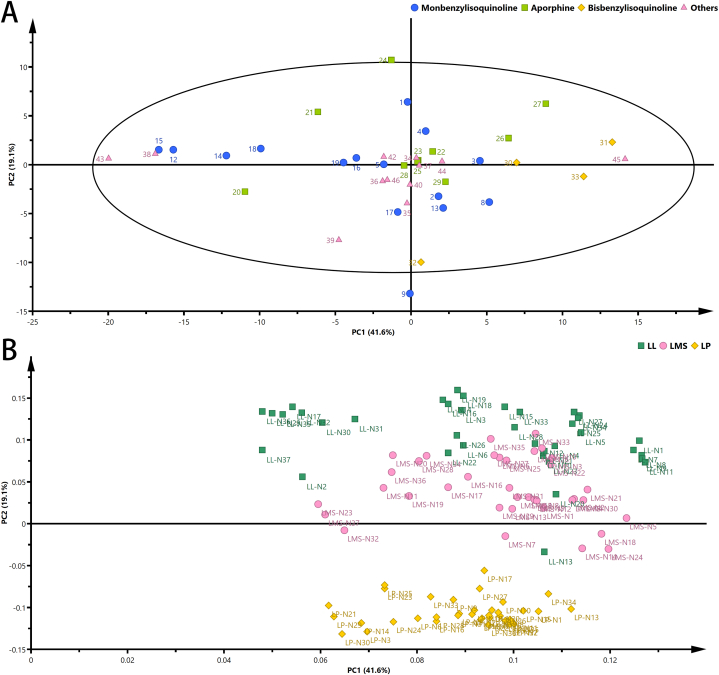
PCA analysis (PC1 and PC2) of alkaloids in the 37 lotus cultivars based on alkaloid content in lotus leaves, milky sap, and seed plumules. A). Score scatter plot. B). Loading plot. Percentages in parentheses represent principal component variance. Numbers in (A) represent the accession number, corresponding to the number in Table 1, Monobenzyl isoquinoline (blue circle), aporphine (green box), bisbenzylisoquinline (yellow diamond), other (pink triangle) alkaloids. Numbers in (B) represent tissue-sample. (For interpretation of the references to colour in this figure legend, the reader is referred to the Web version of this article.)

The accurate masses of 46 BIAs identified in lotus were quantitatively analyzed in lotus leaves, milky sap, and seed plumules. Lotus leaves, milky sap, and seed plumules demonstrated clear separation in the PCA plot by principal component (PC)2 (Fig. 3). There were obvious considerable differences among the different tissues (Fig. 3B), with lotus leaves near the top of the PC2 axis, characterized by high levels of aporphine alkaloids (partitioned as 19.1% of the variance). Lotus milky sap was characterized by lower levels of monobenzylisoquinoline alkaloids and higher levels of bisbenzylisoquinoline alkaloids and other content located in the middle of the PC2 axis. Lotus seed plumules were located in the fourth quadrant and characterized by bisbenzylisoquinoline alkaloids and some monbenzylisoquinoline alkaloids. These alkaloids, including glycosylated and methylated alkaloids, were distinguished by tissue-specific variations (Fig. 3A), probably due to variations in genetic and epigenetic regulation. Furthermore, the plot revealed strong positive correlations among three bisbenzylisoquinoline alkaloids (liensinine [**31**], nelumboferine [**30**], neferine [**33**]), one aporphine alkaloid (nuciferine [**27**]), and one monobenzylisoquinoline alkaloid (isococlaurine [**3**]). Additionally, two glycosylated bisbenzylisoquinoline alkaloids (*N*-methylhigenamine-6-O-Glc [**13**] and *N*-methylisococlaurine-6-O-Glc [**17**]) were clustered away from the other monobenzylisoquinoline alkaloids and were significantly and positively associated with *N*-methylhigenamine (**2**) and armepavine (**8**). These results suggest that these compounds may be associated with seed development because of the epigenetic modification imparting stress resistant [31].

**Fig. 4:**
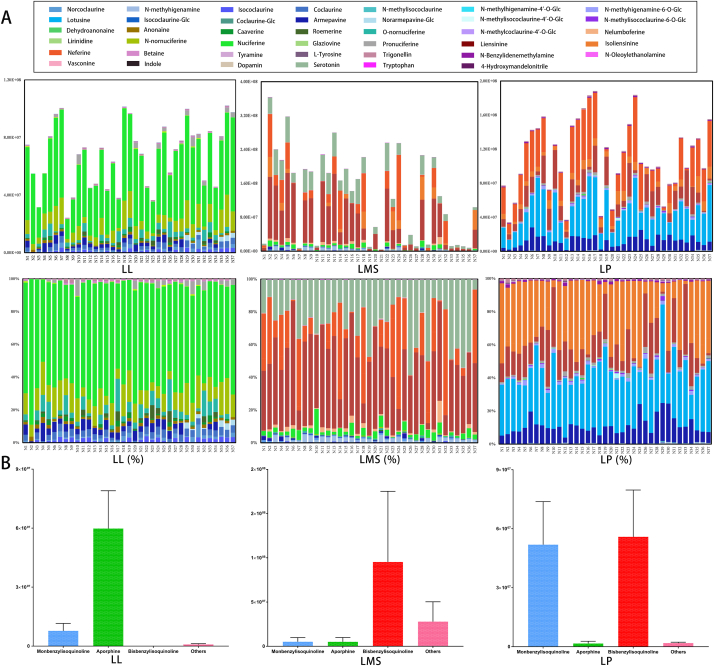
Semiquantification results (A) and relative content (B) of the detected alkaloids in the 37 lotus cultivars.

The BIA content in the three lotus tissues of the 37 lotus cultivars are shown in Fig. 4; nuciferine (**27**), *N*-nornuciferine (**26**), *O*-nornuciferine (**24**), dehydroanonaine (**20**), and caaverine (**22**) were the main compounds in lotus leaves, accounting for >60% of the total BIAs in most of the analyzed cultivars. Aporphine and monobenzylisoquinoline chief were the main alkaloids in lotus leaves (Fig. 4B). Bisbenzylisoquinoline alkaloids (nelumboferine, neferine, liensinine, and isoliensinine) and monobenzylisoquinoline (armepavine, coclaurine, and isococlaurine) alkaloids were the predominant compounds in lotus seed plumules, accounting for >80% of the total BIAs in most of the analyzed cultivars. Liensinine and neferine were the two predominant compounds in lotus milky sap, accounting for nearly 90% or more of the total BIAs in some cultivars for “N1” (Fig. 4A). The contents of the lotus milky sap was more comprehensive determined compared with those of the other tissues and included monobenzylisoquinoline, aporphine, bisbenzylisoquinoline, and other alkaloids. As different alkaloids exhibit different pharmacological and physiological activities, quantitative studies can guide tissue selection in the analyzed cultivars to improve the bioavailability of these alkaloids.

### 3.4 Putative biosynthetic pathway of BIAs

**Fig. 5:**
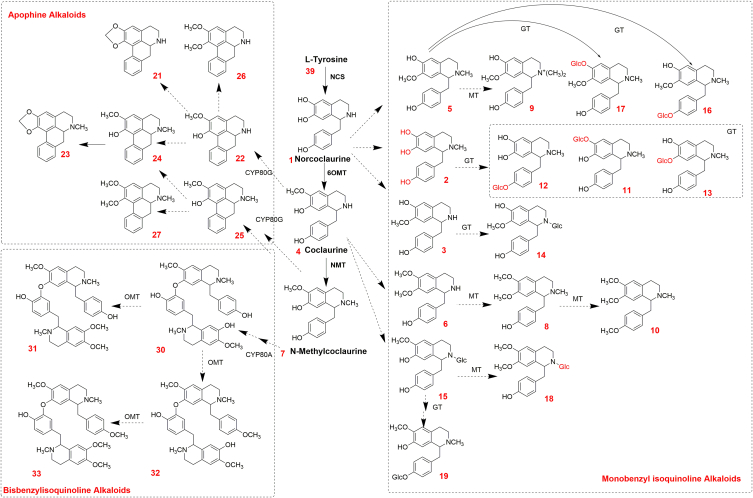
Putative biosynthetic pathway of benzylisoquinoline alkaloids in lotus. The compound numbers are consistent with those in Table 1.

The putative biosynthetic pathway of BIAs was proposed based on the various BIAs detected in lotus leaves, milky sap, and seed plumules (Fig. 5). To the best of our knowledge, these pathways in lotus are yet to be detailed in published reports [8,10]. The results suggest that norcoclaurine, coclaurine, *N*-methylcoclaurine, and reticuline were synthesized from l-tyrosine with the help of *N*-methylstylopine-14-hydroxylase, *O*-methyltransferase, *N*-methyltransferase, cheilanthifoline synthase [27,32,33]. The pathway then divides into three subpathways for the synthesis of bisbenzylisoquinoline alkaloids, aporphine alkaloids, and monobenzylisoquinoline alkaloids. (*S*)-corytuberine synthase (CYP80G) catalyzes the conversion of *N*-methylcoclaurine to generate aporphine alkaloids [28,34]. Furthermore, high levels of five aporphine alkaloids (anonaine, caaverine, *O*-nornuciferine, *N*-nornuciferine, and nuciferine) involved in the aporphine synthesis pathway were detected. The same substrate, *N*-methylcoclaurine, could be used to synthesized bisbenzylisoquinoline alkaloids using berbamunine synthase (CYP80A) [35,36]. Lotusine (**9**) exhibited high positive associations with *N*-methylhigenamine-6-*O*-Glc (**13**, *r* = 0.92) and *N*-methylisococlaurine-6-*O*-Glc (**17**, *r* = 0.81). Norcoclaurine (**1**), isococlaurine (**3**), armepavine (**8**), and lotusine (**9**), which are generated by the biosynthetic pathway of monobenzylisoquinoline alkaloids, also exhibit strong positive correlations (0.5 < *r* < 0.92). In addition, l-tyrosine (**40**), as the primary synthetic precursor of BIAs, was strongly and negatively correlated with coclaurine (**4**, *r* = −0.79) and nuciferine (**27**, *r* = −0.87). Notably, *O*-methyltransferase and *N*-methyltransferase were involved in the modification of benzylisoquinoline alkaloids of the BIAs of lotus. Methylation is involved in the metabolism of most monobenzylisoquinoline alkaloids, usually only once. Glycosylation is another important modification that primarily occurs in lotus seed plumules, mostly after the first methylation of monobenzylisoquinoline alkaloids at the *O*- and *N-*positions.

## 4 Conclusion

Herein, 46 alkaloids, including 9 glycosylated monobenzylisoquinoline alkaloids, were identified using UPLC-QTOF-HRMS and shown to be localized in various tissues (leaves, milky sap, and seed plumules) of sacred lotus using MALDI–MSI. These results directly demonstrate the differences in distribution of these alkaloids in various lotus tissues and further evince the role of secondary metabolites in plant stress resistance. In addition, the glycosylated metabolites were differentially localized, being mostly distributed near the vascular bundles of the lotus seed plumules. These findings lay a foundation for the development and application of natural products for glycosylation. While more information is required to identify the sugar moiety of glycosylated monobenzylisoquinoline alkaloid, the discovery of the glycosylation of monobenzylisoquinoline alkaloids explains the enrichment mechanisms of the different types of naturally glycosylated alkaloids in the different tissues and provides useful insights into functional food and tea development. Furthermore, the content correlations of BIAs among the 37 representative lotus cultivars also provided insights into BIA biosynthesis. To the best of our knowledge, herein, the total alkaloid synthetic pathway in lotus was mapped and the known and possible metabolic pathways were labeled based on quantitative data and reported gene functions for the first time, thus providing new ideas for the further analysis of alkaloid synthetic mechanisms in lotus and BIA biosynthesis. Furthermore, the quantitative data serves as a scientific reference for planting selection, and the development and application of different varieties of lotus as well as provides guidance for lotus breeding.

## Declarations

### Author contribution statement

Chenyang Hao, Wei Yang, Yuetong Yu, Yan Liu, Xiaolu Wei and Sha Chen: Conceived and designed the experiments; Analyzed and interpreted the data.

Gangqiang Dong, Yongping Zhu and Xiaolu Wei: Contributed reagents, materials, analysis tools or data.

Chenyang Hao, Jun Zhang and Sha Chen: Performed the experiments; Wrote the Paper.

### Data availability statement

Data included in article/supplementary material/referenced in article.

## Declaration of competing interest

The authors declare that they have no known competing financial interests or personal relationships that could have appeared to influence the work reported in this paper.
