# Supplementary material for: Visualization and identification of benzylisoquinoline alkaloids in various nelumbo nucifera tissues
Source: Heliyon. 2023 May 19;9(6):e16138. doi: 10.1016/j.heliyon.2023.e16138 (PMC10220311; doi:10.1016/j.heliyon.2023.e16138)

Table S1 The information of 37 *Nelumbo* cultivars

| Number | Name |
| --- | --- |
| N1 | MZH |
| N2 | MLL |
| N3 | QLHL |
| N4 | BLFR |
| N5 | SCDH |
| N6 | SF |
| N7 | TK58 |
| N8 | XHHL |
| N9 | XHY |
| N10 | BZGY |
| N11 | XLZ13 |
| N12 | YCCN |
| N13 | YO |
| N14 | ZBFHL |
| N15 | ZYSZ |
| N16 | ZY |
| N17 | CJB |
| N18 | CZBL |
| N19 | CHQY |
| N20 | CYB |
| N21 | DZY |
| N22 | DFH2 |
| N23 | ECHL |
| N24 | GQH |
| N25 | HXYR |
| N26 | Ti-12 |
| N27 | BJ |
| N28 | BiJ |
| N29 | GCCTBL |
| N30 | HLJ |
| N31 | JX35 |
| N32 | SH |
| N33 | RYH |
| N34 | TGHQL |
| N35 | SLH |
| N36 | JX21 |
| N37 | JFR1 |

Supplement Figure 1: A LC-MS chromatogram of a standard solution mixture.


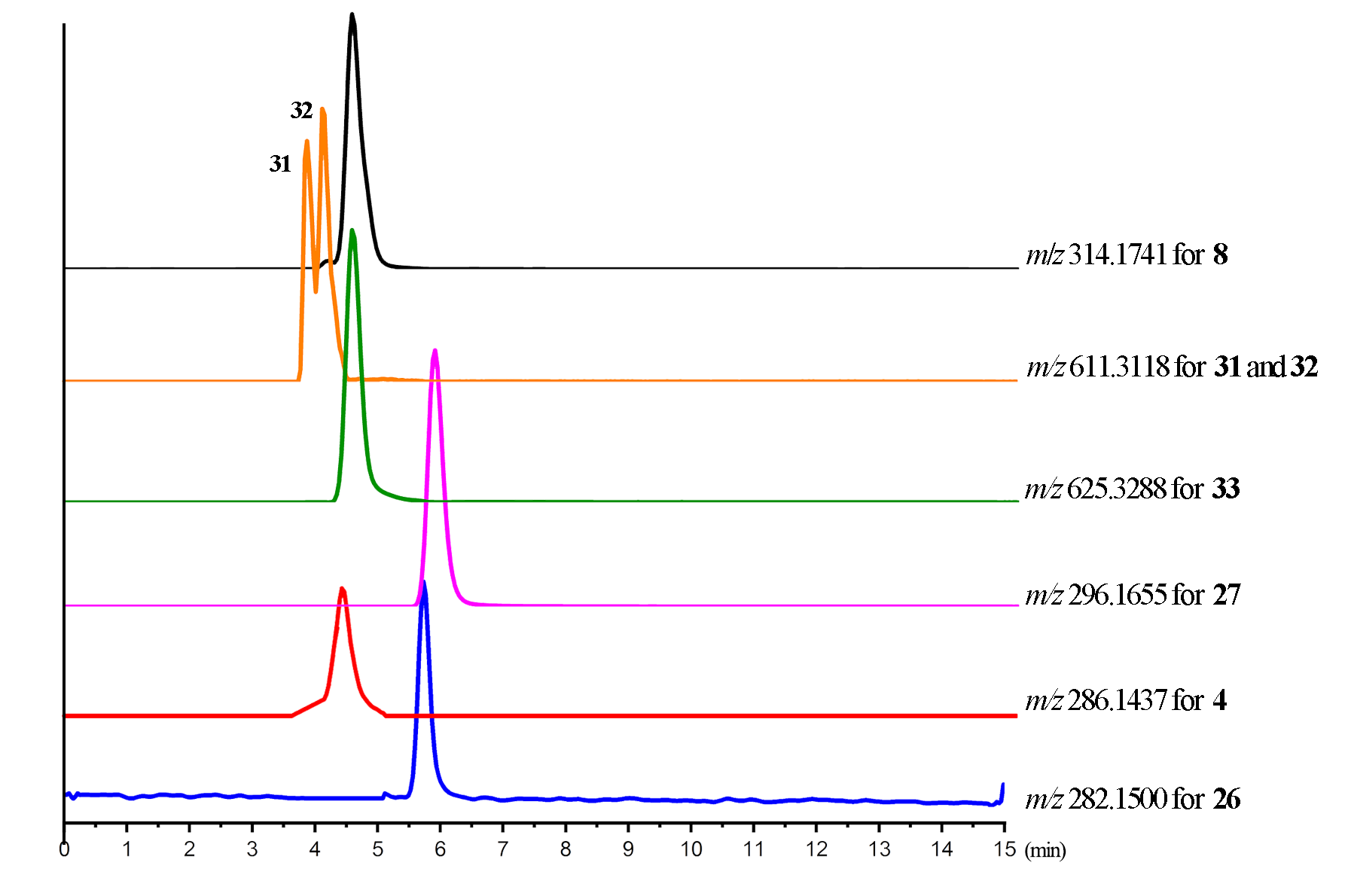


Supplement Figure 2: A LC-MS chromatogram of newly annotated chemicals.


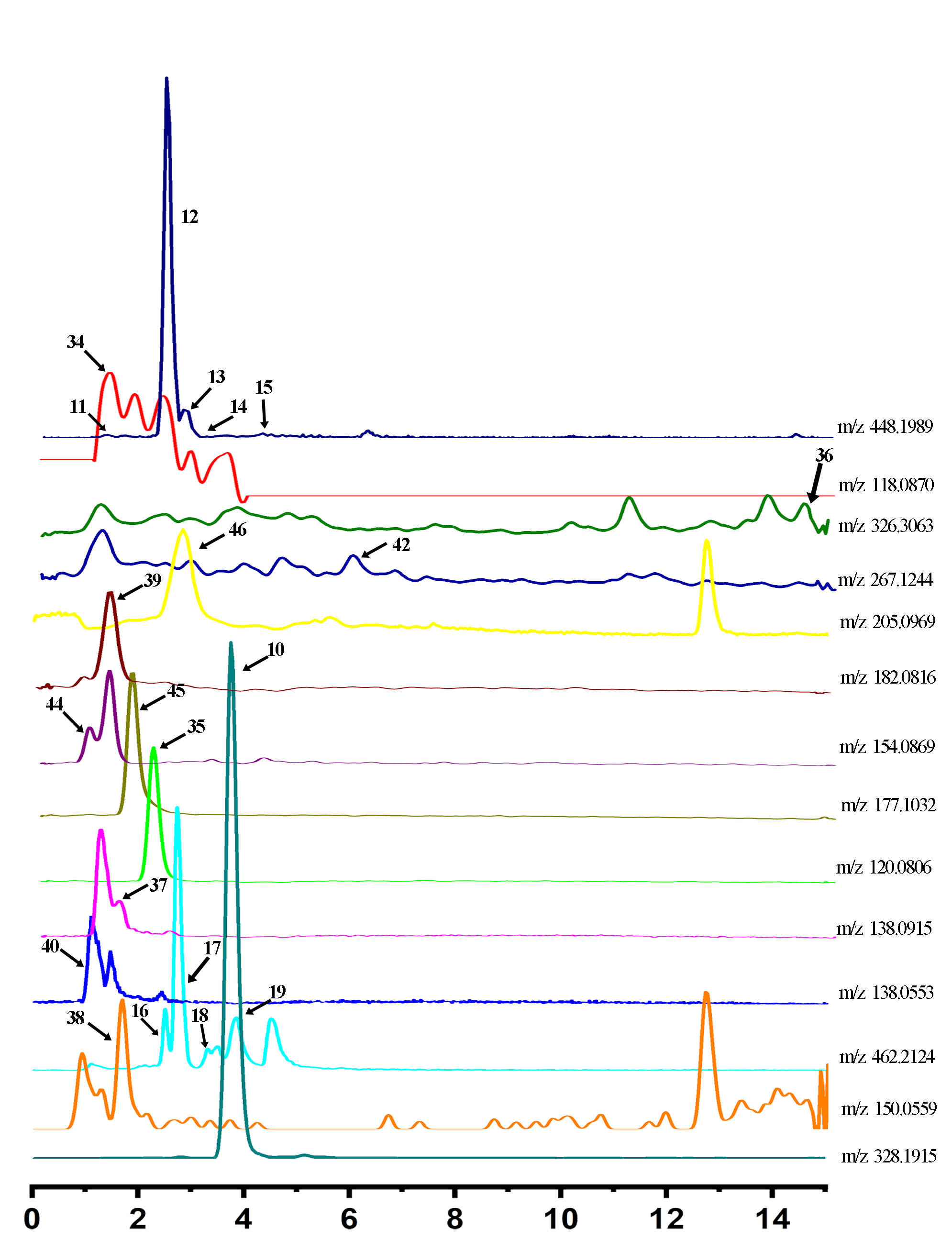

Supplement: Multimedia component 1 [file mmc1.docx]
